# Genome sequences of two novel phages infecting marine roseobacters

**DOI:** 10.1111/j.1462-2920.2009.01927.x

**Published:** 2009-08

**Authors:** Yanlin Zhao, Kui Wang, Nianzhi Jiao, Feng Chen

**Affiliations:** 1 Center of Marine Biotechnology, University of Maryland Biotechnology InstituteBaltimore, MD 21202, USA; 2 State Key Laboratory of Marine Environmental Science, Xiamen UniversityXiamen 361005, China

## Abstract

Two bacteriophages, DSS3Φ2 and EE36Φ1, which infect marine roseobacters *Silicibacter pomeroyi* DSS-3 and *Sulfitobacter* sp. EE-36, respectively, were isolated from Baltimore Inner Harbor water. These two roseophages resemble bacteriophage N4, a large, short-tailed phage infecting *Escherichia coli* K12, in terms of their morphology and genomic structure. The full genome sequences of DSS3Φ2 and EE36Φ1 reveal that their genome sizes are 74.6 and 73.3 kb, respectively, and they both contain a highly conserved N4-like DNA replication and transcription system. Both roseophages contain a large virion-encapsidated RNA polymerase gene (> 10 kb), which was first discovered in N4. DSS3Φ2 and EE36Φ1 also possess several genes (i.e. ribonucleotide reductase and thioredoxin) that are most similar to the genes in roseobacters. Overall, the two roseophages are highly closely related, and share 80–94% nucleotide sequence identity over 85% of their ORFs. This is the first report of N4-like phages infecting marine bacteria and the second report of N4-like phage since the discovery of phage N4 40 years ago. The finding of these two N4-like roseophages will allow us to further explore the specific phage–host interaction and evolution for this unique group of bacteriophages.

## Introduction

The *Roseobacter* lineage in α-*Proteobacteria* comprises up to 25% of the bacterial community in seawater (Wagner-Döbler and Biebl, 2006). Roseobacters are diverse and ubiquitous in marine environments, and play an important role in marine biogeochemical cycles (Buchan *et al*., 2005; Wagner-Döbler and Biebl, 2006; Moran *et al*., 2007). Due to their ecological relevance, complete or draft genome sequences for more than 40 marine roseobacters are available (Brinkhoff *et al*., 2008). Recently, phage-like gene transfer agents (Lang and Beatty, 2007; Paul, 2008) and inducible prophages (Chen *et al*., 2006) have been found in *Roseobacter* genomes, indicating that virus-mediated gene transfer could be an important driving force for their genomic diversification and ecological adaptation.

Currently, only one lytic phage (SIO1), which infects a marine roseobacterium (*Roseobacter* sp. SIO67) has been reported (Rohwer *et al*., 2000). SIO1 is a T7-like podovirus containing the T7-like DNA replication genes and the genes involved in phosphate metabolism. To date, the vast majority of known marine podoviruses are the members of the T7 supergroup (i.e. P60, SIO1, P-SSP7, Syn5, S-CBP1, S-CBP2 and S-CBP3) (Rohwer *et al*., 2000; Chen and Lu, 2002; Sullivan *et al*., 2005; Pope *et al*., 2007; Wang and Chen, 2008), and they all contain a conserved DNA polymerase in their replication module (Wang and Chen, 2008).

*Silicibacter pomeroyi* DSS-3 and *Sulfitobacter* sp. EE-36 are among those roseobacters whose genomes have been sequenced. Both strains were isolated from Georgia coastal waters*. Silicibacter pomeroyi* DSS-3, the first roseobacterium with a sequenced genome, has been served as a model organism for studying the eco-physiological strategies of heterotrophic marine bacteria (Moran *et al*., 2004; Bürgmann *et al*., 2007). *Sulfitobacter* sp. EE-36 has a high inorganic sulfur oxidation activity and has been a model organism for studying sulfur cycle in coastal environments (Roseobase: http://www.roseobase.org/).

In a study undertaken to isolate bacteriophages from marine roseobacters, two novel phages (not seen in known marine phages) were isolated from *S. pomeroyi* DSS-3 and *Sulfitobacter* sp. EE-36 respectively. Here, we report the morphology, basic biology and genome sequences of these two newly discovered roseophages.

## Results and discussion

### Morphology and basic biology of DSS3Φ2 and EE36Φ1

**Fig. 1:**
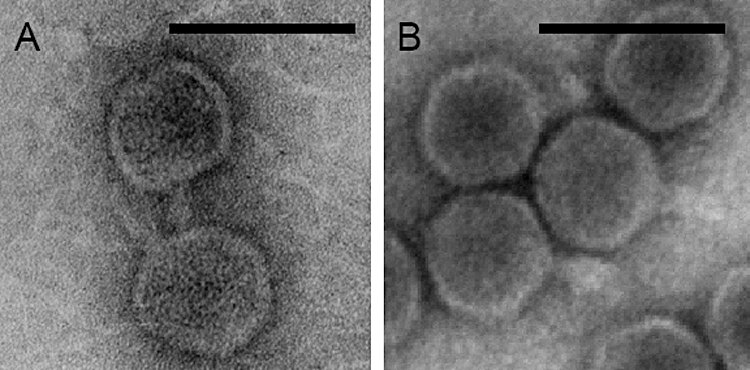
Transmission electron microscopy images of roseophages. (A) DSS3Φ2. (B) EE36Φ1. Scale bar = 100 nm.

**Fig. 2:**
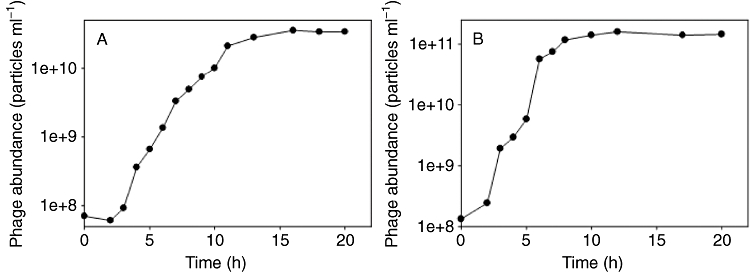
One-step growth curves of roseophages. (A) DSS3Φ2. (B) EE36Φ1.

Phages DSS3Φ2 and EE36Φ1 were isolated from Baltimore Inner Harbor Pier V using an enrichment method. Both phages formed clear plaques. DSS3Φ2 produced large, clear plaques with irregular edges, while EE36Φ1 produced small, clear, round plaques. DSS3Φ2 and EE36Φ1 infected only *S. pomeroyi* DSS-3 and *Sulfitobacter* sp. EE-36, respectively, and did not cross-infect 13 other diverse marine *Roseobacter* strains (listed in the *Experiment procedures*). These two phages are morphologically similar to each other with icosahedral capsids (∼70 nm in diameter) and visible short tails (∼26 nm long) (Fig. 1). The capsids of DSS3Φ2 and EE36Φ1 are larger than those of T7-like podoviruses. The tails of DSS3Φ2 and EE36Φ1 are longer that those of T7-like podoviruses, but much shorter than those of typical myoviruses and siphoviruses. Morphologically, they resemble coliphage N4 (with a capsid size of ∼70 nm) (Kazmierczak and Rothman-Denes, 2006), a unique phage isolated from a sewage source in 1960s (Schito *et al*., 1967). The phage family *Podoviridae* currently consists of four genera (T7-like, Φ29-like, P22-like, N4-like), and phage N4 is the only member within N4-like genus (http://www.ncbi.nlm.nih.gov/ICTVdb/Ictv/index.htm). The infectivities of the DSS3Φ2 and EE36Φ1 were not affected by chloroform treatment (2%), indicating that neither of them is membrane-coated. Both phages had a prolonged lysis period. The latent periods of DSS3Φ2 and EE36Φ1 were about 3 and 2 h, respectively, followed by a gradual increase of released viral particles (Fig. 2A and B). It took about 15 and 10 h for DSS3Φ2 and EE36Φ1, respectively, to reach their growth plateaus and this resulted in the burst sizes approximately 350 and 1500 viral particles respectively. Delayed lysis and large burst size were also found in phage N4. A single N4-infected *Escherichia coli* produces *c*. 3000 viruses 3 h post infection (Schito, 1974). It is noteworthy that *S. pomeroyi* DSS-3 and *Sulfitobacter* sp.EE-36 grow nearly four times slower than *E. coli*, and this may partially explain the longer lysis period of these two roseophages compared with N4.

### General genomic features of DSS3Φ2 and EE36Φ1

DSS3Φ2 and EE36Φ1 are linear, double-stranded DNA viruses. The genome sizes of DSS3Φ2 and EE36Φ1 are 74.6 and 73.3 kb, respectively, much larger than most known podoviruses, such as T7-like (37–46 kb), P22-like (39–50 kb) and Φ29-like (11–21 kb) podoviruses (http://www.ncbi.nlm.nih.gov/genomes/GenomesGroup.cgi?taxid=28883), but comparable to phage N4 (*c*. 70 kb, NCBI Accession No. EF056009). The G+C contents of DSS3Φ2 and EE36Φ1 genomes are 47.9% and 47.0%, respectively, higher than that of phage N4 (41%) but much lower than those of their hosts *S. pomeroyi* DSS-3 (64%) and *Sulfitobacter* sp. EE-36 (60%). A total of 81 open reading frames (ORFs) were identified in DSS3Φ2 and 79 ORFs were identified in EE36Φ1.

**Fig. 3:**
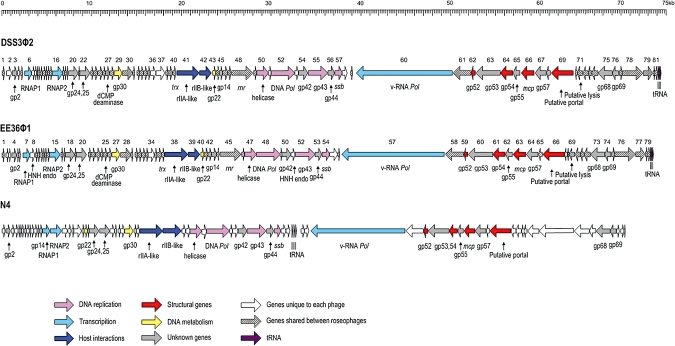
Genome organization and comparison of roseophages DSS3Φ2 and EE36Φ1 and coliphage N4. ORFs are depicted by leftward or rightward oriented arrows according to the direction of transcription. For comparison, the roseophage ORFs similar to N4 were named based on the N4 annotation.

Despite their different host origin, the two roseophages share approximately 85% ORFs (70 ORFs) and have similar overall genome organization (Fig. 3). The ORFs shared between these two phages are 80–94% identical at the DNA level, and 83–98% identical at the amino acid level. The ORFs unique to each phage are mostly distributed on the left half side of their genomes (Fig. S1). Among all the identified ORFs, 26 ORFs from both roseophages are most closely related to genes from N4, with 26–57% amino acid identity, accounting for *ca*. 30% of both roseophages genomes (Table 1). Roseophages and N4 share DNA metabolism and replication genes, transcription genes, structural genes, host interaction genes and some additional genes without known function. DSS3Φ2 and EE36Φ1 also contain ORFs similar to genes from other types of phages and bacteria (Table 1), indicating the mosaic feature of the phage genomes. Approximately 40% of both roseophages ORFs have no matches in the database.

**Table 1:** Major predicted ORFs of roseophages DSS3Φ2 and EE36Φ1, the shaded ORFs are those similar to N4 genes.

ORF no.	DSS3Φ2 (aa)	ORF no.	EE36Φ1 (aa)	Best hit	Potential function	% aa identity (similarity)^a^
3	156	4	172	N4 gp2	Unknown	28 (43)
6	260	7	260	N4 gp15, RNA polymerase subunit 1	Middle genes transcription	39 (61)
16	401	15	401	N4 gp16, RNA polymerase subunit 2	Middle genes transcription	39 (52)
20	398	18	398	N4 gp24, putative ATPase, AAA superfamily	Unknown	31 (49)
22	393	20	388	N4 gp25, hypothetical protein, vWFA Super-family	Unknown	26 (43)
23	92	21	92	α-*proteobacter Bradyrhizobium* sp. ORS278, hypothetical protein	Unknown	42 (63)
27	142	25	142	Bacteriophage Phi JL001 gp30, putative deoxycytidylate deaminase	DNA metabolism	46 (60)
28	142	26	143	*Mycobacterium* phage Omega, gp174	Unknown	28 (51)
29	282	27	293	N4 gp30, putative thymidylate synthase	DNA metabolism	39 (52)
30	383	28	383	*Erwinia amylovora* phage Era103, hypothetical protein g26	Unknown	27 (43)
31	73	29	73	*Shewanella oneidensis* MR-1, hypothetical protein SO_2678	Unknown	53 (65)
32	110	30	110	*Roseobacter* sp. AzwK-3b, hypothetical protein	Unknown	34 (51)
33	166	31	166	*Acidovorax avenae* ssp. *citrulli* AAC00-1, hypothetical protein	Unknown	36 (50)
34	168	32	168	*Delftia acidovorans* SPH-1, hypothetical protein	Unknown	52 (69)
40	105	37	105	*Paracoccus denitrificans* PD1222, thioredoxin	DNA metabolism	33 (64)
41	862	38	860	N4 gp33, rII A-like protein	Lysis inhibition	35 (49)
42	476	39	476	N4 gp34, rII B-like protein	Lysis inhibition	30 (48)
43	102	40	102	N4 gp22, putative homing endonuclease	DNA metabolism	52 (69)
45	129	42	129	N4 gp14	Unknown	51 (65)
46	181	43	182	*Azoarcus* sp. EbN1, potential phage protein	Unknown	43 (52)
48	776	45	776	*Roseovarius* sp. HT2601, ribonucleoside-diphosphate reductase	DNA metabolism	56 (70)
50	425	47	425	N4 gp37, DNA helicase	DNA replication	30 (53)
52	876	48	876	N4 gp39, DNA polymerase	DNA replication	54 (70)
						44 (60)
54	331	50	331	N4 gp42	Unknown	44 (65)
55	730	52	731	N4 gp43	DNA replication	46 (66)
56	244	53	244	N4 gp44	Unknown	46 (66)
57	277	54	277	N4 gp45, DNA replication	Single-stranded DNA binding protein	32 (48)
60	3632	57	3786	N4 gp50, virion-encapsulated RNA polymerase	Early genes transcription	27 (46)
61	666	58	672	*Roseobacter* phage SIO1 gp24, C-Terminal partial sequence	Unknown	41 (60)
62	148	59	133	N4 gp52, C-Terminal partial sequence	Structural gene	40 (60)
63	915	60	913	N4 gp53	Unknown	29 (47)
64	450	61	452	N4 gp54	Structural gene	44 (61)
65	251	62	249	N4 gp55	Unknown	42 (61)
66	482	63	482	N4 gp56, major coat protein	Structural gene	47 (63)
67	447	64	448	N4 gp57	Unknown	36 (57)
69	800	66	800	N4 gp59, putative portal gene	Structural gene	45 (65)
71	190	69	190	*Roseobacter* sp. CCS2, hypothetical protein	Putative lysis gene	47 (61)
75	538	73	538	N4 gp68	Unknown	57 (73)
76	229	74	229	N4 gp69	Unknown	42 (69)
81	115	79	115	*E. coli* SE11 hypothetical protein ECSE_1686	Unknown	39 (54)

a Most ORFs are similar between DSS3Φ2 and EE36Φ1, therefore only the best hits to DSS3Φ2 are listed here.

### A large virion-encapsidated RNA polymerase gene

Strikingly, both DSS3Φ2 and EE36Φ1 contain a large ORF, which flanks more than 10 kb of phage genome (Table 1, Fig. 3). Those ORFs can be translated into 3632 aa and 3786 aa proteins for DSS3Φ2 and EE36Φ1, respectively, which match a 3500 aa virion-encapsidated RNA polymerase (vRNAP) in phage N4 (27% amino acid identity). Among all the known phages, N4 is the only one that contains this super large vRNAP gene (Falco *et al*., 1980). It is intriguing that these phages carry a conserved gene that makes up one-seventh of their genome. N4 vRNAP is responsible for early transcription (Falco *et al*., 1977) and DNA replication (Kazmierczak and Rothman-Denes, 2006). N4 vRNAP is packed in viral particles and injected into host cell upon infection (Falco *et al*., 1977; 1980). Similar to N4 vRNAP, the two roseophage vRNAPs do not contain any cysteine residues. The lack of cysteine residues could be important for v-RNAP to enter the host cells (Kazmierczak *et al*., 2002). Four conserved T7-like RNAP motifs (motifs T/DxxGR, A, B and C) and their catalytic residues (R424, K670, Y678, D559 and D951) described in N4 vRNAP (Kazmierczak *et al*., 2002) are also present in the two roseophages (Fig. S2), suggesting the similar function of vRNAP in DSS3Φ2, EE36Φ1 and N4. The advantage of a > 10 kb RNA polymerase gene to a phage is still not clear. However, it is interesting that this unique feature is conserved among the N4-like phages.

The two roseophages also encode two different RNA polymerase subunits (RNAP1 and RNAP2) which are similar to N4 RNAP1 and RNAP2. RNAP1 and RNAP2 constitute N4 RNA polymerase II (RNAP II) (Willis *et al*., 2002). RNAP II together with gp2, which also appears in roseophages, activates the transcription of N4 middle genes (Willis *et al*., 2002). In DSS3Φ2 and EE36Φ1, there are several small insertions (∼2 kb) between the RNAP1 and RNAP2 (Fig. 3). However, there is only a 47 bp gap between N4 RNAP1 and RNAP2. The presence of vRNAP and RNAP II in roseophages suggests that these two roseophages may use similar early and middle transcription machinery to that in N4.

### Conserved DNA replication module

**Fig. 4:**
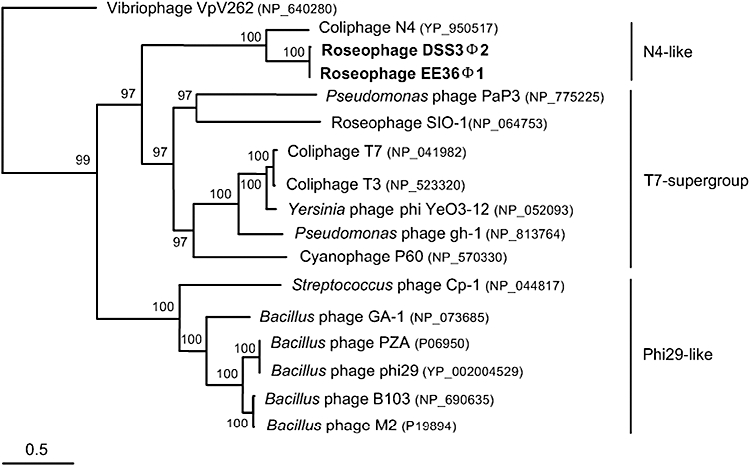
Neighbour-joining tree constructed based on the aligned DNA *pol* amino acid sequences of 17 podoviruses. Phage VpV262 was used as an outgroup. The scale bar represents 0.5 fixed mutations per amino acid position. Bootstrap = 1000.

Both DSS3Φ2 and EE36Φ1 possess the complete N4-like DNA replication system, including DNA helicase, DNA polymerase (DNA *pol*), single-stranded DNA binding protein (*ssb*), gp43 and vRNAP, and the relative gene order of these DNA replication genes is conserved among the two roseophages and N4 (Fig. 3). These genes are essential for *in vivo* N4 DNA replication (Kazmierczak and Rothman-Denes, 2006). The DNA *pol* genes of DSS3Φ2 and EE36Φ1 share high amino acid identity (53%) with N4 DNA *pol* (Table 1). DNA *pol* of the two roseophages and N4 all contain the DNA polymerase A domain and 3′-5′ exonuclease domain but lacks 5′-3′ exonuclease domain (Kazmierczak and Rothman-Denes, 2006). DNA *pol* gene phylogenetic analysis shows that DSS3Φ2 and EE36Φ1 are closely related to N4, but distantly related to T7 supergroup podoviruses (Fig. 4). When the N4-like DNA *pol* sequences were searched against the metagenomic databases, a limited amount of environmental sequences (11 hits) were obtained (Table 2). Interestingly, all the close hits were found in the nearshore GOS sites (i.e. harbors, basins, or mangrove-associated habitats), but not in the open ocean sites. In addition, we also searched the GOS database using other N4 like genes from DSS3Φ2 and EE36Φ1 genomes. Seventeen of 26 genes from these two roseophages could find homologous sequences in 13 GOS sites, resulting in a total of 96 N4-like environmental sequences (data not shown). Among these sequences, only two were found in the open ocean, and the rest 94 sequences were from coastal sites. These results suggest that N4-like roseophages are more abundant in the coastal waters than the open ocean water. It has been known that roseobacters are more abundant in the coastal water compared with the oceanic water (Buchan *et al*., 2005). Whether the geographic pattern of N4-like phages could be related to the distribution of roseobacters warrants further study.

**Table 2:** Homologues of N4-like DNA *pol* from GOS database.

Location	Length (aa)	% aa identity (similarity) to N4 DNA *pol*	Temperature (°C)	*E*-value	CAMERA accession number
GS003 Browns Bank, Gulf of Maine (Coastal)	241	43 (61)	11.7	3e^−40^	JCVI_PEP_1105101727847
GS005 Bedford Basin, Nova Scotia (Coastal)	292	44 (65)	15	3e^−63^	JCVI_PEP_1105130909873
GS005 Bedford Basin, Nova Scotia (Coastal)	331	29 (48)	15	3e^−33^	JCVI_PEP_1105128486423
GS005 Bedford Basin, Nova Scotia (Coastal)	361	29 (48)	15	3e^−33^	JCVI_PEP_1105132655935
GS005 Bedford Basin, Nova Scotia (Coastal)	323	31 (50)	15	6e^−31^	JCVI_PEP_1105124722171
GS008 Newport Harbor, RI (Coastal)	365	51 (71)	9.4	2e^−107^	JCVI_PEP_1105108615979
GS008 Newport Harbor, RI (Coastal)	291	61 (77)	9.4	5e^−93^	JCVI_PEP_1105142578775
GS009 Block Island, NY (Coastal)	135	58 (75)	11	3e^−29^	JCVI_PEP_1105118337801
GS009 Block Island, NY (Coastal)	203	51 (66)	11	1e^−45^	JCVI_PEP_1105120605863
GS032 Mangrove on Isabella Island (Mangrove)	302	52 (71)	25.4	4e^−77^	JCVI_PEP_1105137183151
GS032 Mangrove on Isabella Island (Mangrove)	223	59 (74)	25.4	2e^−68^	JCVI_PEP_1105112552903

Both roseophages possess a single-stranded DNA-binding protein (*ssb*) homologous to N4 *ssb*. N4 *ssb* belongs to a novel protein family and is involved in DNA replication, DNA recombination (Lindberg *et al*., 1989; Choi *et al*., 1995) and activation of *E. coli* RNA polymerase at late N4 transcription (Cho *et al*., 1995). The function of roseophages *ssb* genes is not clear. Previous research suggested that the single-stranded DNA binding activity and transcriptional activation of N4 *ssb* are separable, and the residues S260, K264 and K265 in the C-terminal of N4 *ssb* constitute part or all of an ‘activating region’ required for transcriptional activation, and were proposed to activating region (Miller *et al*., 1997). However, alignment of the amino acids of the *ssb* from roseophages and N4 shows that the C-terminal of roseophages *ssb* do not contain Lys residues at the end of polypeptide (Fig. S3). Moreover, the *ssb* residue Y75, which is essential for ssDNA binding activation in N4 was also not found in DSS3Φ2 and EE36Φ1. Further study on roseophages *ssb* could provide a better understanding on the function of these binding activity sites.

### Roseophages contain host-related genes

Aside from the N4-like genes, DSS3Φ2 and EE36Φ1 also contain certain *Roseobacter*-related genes, suggesting that genetic exchange occurred between roseobacters and their phages. For examples, the roseophage ribonucleotide reductase (*rnr*) genes share the highest amino acid identity (56%) with nucleotide reductase from their *Roseobacter* hosts. The *rnr* phylogenetic analysis shows that DSS3Φ2 and EE36Φ1 cluster together with roseobacters (Fig. S4). Interestingly, N4 does not contain this *rnr* gene. The *rnr* converts ribonucleosides to deoxynucleotide, and is a key enzyme involved in DNA synthesis (Jordan and Reichard, 1998). The *rnr* gene is rarely seen in non-marine podoviruses, such as T7, T3 and P22. However, several podoviruses isolated from marine environments, such as SIO1, P60, P-SSP7 and syn5 contain *rnr* (Rohwer *et al*., 2000; Chen and Lu, 2002; Sullivan *et al*., 2005; Pope *et al*., 2007). Perhaps, obtaining sufficient free nucleotides for phage DNA synthesis is critical in the phosphorus-limited marine environment (Chen and Lu, 2002; Sullivan *et al*., 2005). Viral metagenomic analysis has shown that *rnr* is among the most abundant genes found in Sargasso Sea (Angly *et al*., 2006).

DSS3Φ2 and EE36Φ1 both contain a thioredoxin (*trx*) gene (105 aa) that shares high sequence homology with bacterial *trx* (Table 1). When binding with host thioredoxin, T7 DNA polymerase could increase its processing speed (Mark and Richardson, 1976; Huber *et al*., 1987). Thioredoxin has been found in other T7-like marine phage genomes (Rohwer *et al*., 2000; Chen and Lu, 2002; Pope *et al*., 2007) and appears to be a universal accessory cofactor of the replication module in these marine phages (Hardies *et al*., 2003). In contrast, neither coliphage N4 nor T7 contains *trx*. The finding of host-related thioredoxin in marine roseophages and other marine phages suggests that *trx* might be important to the phage survival in marine environments.

### tRNA in roseophages

Using tRNA scan-SE, three tRNA genes were identified in both DSS3Φ2 and EE36Φ1 genomes (Table 3). DSS3Φ2 and EE36Φ1 both encode the tRNA gene CCA (Pro) and TCA (Ser). In hosts DSS-3 and EE-36, CCA and TCA are the rarest codons that code for Pro and Ser respectively. In contrast, CCA and TCA are abundant codes for Pro and Ser in phages DSS3Φ2 and EE36Φ1 (Table 3). It is possible that these two tRNAs are important for the roseophages during their translation stage (Bailly-Bechet *et al*., 2007). However, the reason for the presence of tRNA ATG (Met) in DSS3Φ2 and ATC (Ile) in EE36Φ1 is unclear.

**Table 3:** Codon usage in phage DSS3Φ2, EE36Φ1 and their host strains (DSS-3 and EE-36), the codon in bold indicates the codon encoded by roseophages tRNAs.

		Usage frequency: per thousand (number)				Usage frequency: per thousand (number)	
Codon	Amino acid	DSS3Φ2	DSS-3	Codon	Amino acid	EE36Φ1	EE-36^a^
CCT	Pro	12.7	2.8	CCT	Pro	18.7	4.3
CCC	Pro	6.9	20.8	CCC	Pro	6.9	19.2
**CCA**	Pro	17.1	2.6	**CCA**	Pro	10.3	2.1
CCG	Pro	7.3	25.8	CCG	Pro	7.1	26.7
TCT	Ser	22.8	2.1	TCT	Ser	19.6	0
TCC	Ser	15.8	8.2	TCC	Ser	8.0	4.3
**TCA**	Ser	25.8	1.8	**TCA**	Ser	11.4	0
TCG	Ser	16.5	20.1	TCG	Ser	8.2	12.8
**ATG**	Met	26.6	27.5	ATT **ATC**ATA	Ile Ile Ile	22.5 32.4 1.4	9.6 52.4 0

a Analysed based on one contig for EE-36.

### DNA metabolism genes

Both roseophages encode a putative deoxycytidylate deaminase (dCMP deaminase) gene, which is most similar to the dCMP deaminase in phage Phi JL001 (Lohr *et al*., 2005) (Table 1). Deoxycytidylate deaminase catalyses the dCMP to dUMP. The roseophages also code for putative thymidylate synthase (thyX) protein, which generates thymidine monophosphate (dTMP) from deoxyuridine monophosphate (dUMP). Both roseophages encode a putative HNH endonuclease homologous to N4 gp22 (ORF 43 in DSS3Φ2, and ORF40 in EE36Φ1). Compared with DSS3Φ2, EE36Φ1 encodes two additional endonucleases (ORF 8 and ORF 51) (Table 1, Fig. 3).

### Lysis gene

It is noteworthy that homologue of N4 lysis gene, a new family of murein hydrolase (Stojković and Rothman-Denes, 2007), was not detected in DSS3Φ2 or EE36Φ1 genomes. However, a hypothetical protein (190 aa, ORF 71 in DSS3Φ2 and ORF 69 in EE36Φ1) located in the late region of DSS3Φ2 and EE36Φ1 genomes likely act as a lysis gene because this protein is similar to a lytic enzyme found in roseobacterium *Sagittula stellata* E-37 (37% amino acid identity).

### Structural genes

Four structural genes are shared between roseophages and N4 (Table 1, Fig. 3). Both DSS3Φ2 and EE36Φ1 encode the major capsid protein (*mcp*) and putative phage portal gene, which are similar to their N4 counterparts (Table 1). The homologues of N4 structural genes gp52 and gp54 were also identified in the roseophages (Choi *et al*., 2008).

## Conclusions

Phage N4 has been studied for 40 years without a comparable system. The genome sequences of DSS3Φ2 and EE36Φ1 reveal a close relationship between coliphage N4 and these two roseophages. Discovery of the two N4-like marine phages serves as a good reference system for further understanding of phage biology and evolution. The two podovirus-like roseophages are distantly related to all the known marine podoviruses. DSS3Φ2 and EE36Φ1 are warranted to investigate the ecological role of N4-like phage on marine roseobacters.

## Experimental procedures

### Roseobacter strains

*Silicibacter pomeroyi* DSS-3 and *Sulfitobacter* sp. EE-36 were kindly provided by Dr M.A. Moran at the University of Georgia. Bacterial strains were grown in YTSS medium (4 g l^−1^ yeast extract, 2.5 g l^−1^ tryptone, 20 g l^−1^ Crystal Sea) at 28°C.

### Isolation of roseophages

Water samples were collected from Baltimore Inner Harbor Pier V on 24 January 2007, and immediately filtered through 0.22 μm polycarbonate membrane filters (Millipore, Bedford, MA, USA). Filtrate of 100 ml was added to 150 ml of exponentially growing bacterial cultures and incubated overnight. Cultures were then centrifuged at 10 000 *g* for 20 min to remove bacterial cells. Cell-free lysates of 10–100 μl were added into 1 ml of exponentially growing cultures, and plated using plaque assay according to a protocol described elsewhere (Suttle and Chen, 1992). Each phage isolate was purified at least three times by plaque assay.

### Transmission electron microscopy

One drop of purified roseophage particles was adsorbed to the 200-mesh Formvar/carbon-coated copper grid for several minutes and then the grids were stained with 0.5% aqueous uranyl acetate for *c*. 30 s. Samples were examined with a Zeiss CEM902 transmission electron microscope operated at 80 kV (University of Delaware, Newark). Images were taken using a Megaview II digital camera (Soft Imaging System, Lakewood, CO).

### Cross-infection

The cross-infectivities of roseophages were tested against other marine *Roseobacter* strains. The 13 tested *Roseobacter* strains are *Roseovarius nubinhibens* ISM, *Sulfitobacter* 1921, *Roseobacter* sp. TM1038, *Silicibacter* sp. TM1040, *Roseobacter* sp. TM1039, *Roseovarius* sp. TM1042, *Phaeobacter* 27–4, *Roseobacter denitrificans* ATCC 33942, *Roseobacter litoralis* ATCC 49566, *Sulfitobacter* strain CHSB4, JL351, T11, and CBB406. Strains DSS-3, EE-36 and ISM were provided by M.A. Moran, while strains TM1038, TM1039, TM1040, TM1042, *Phaeobacter* strain 27-4, *Roseobacter denitrificans* ATCC 33942, and *Roseobacter litoralis* ATCC 49566 were provided by R. Belas at University of Maryland Biotechnology Institute (UMBI). The remaining strains were isolated in our laboratories. Exponentially growing cultures of these bacteria strains were incubated with roseophage lysates for 30 min and then plated using plaque assay.

### Growth curve experiments

Exponentially growing cultures of *S. pomeroyi* DSS-3 and *Sulfitobacter* sp. EE-36 (100 ml) were inoculated with the DSS3Φ2 and EE36 Φ1 at a multiplicity of infection (moi) of 0.1. After inoculation, an aliquot of the cell suspension was collected from each culture every 1 h for 20 h, and the numbers of DSS3Φ2 and EE36 Φ1 were determined by epifluorescence microscopic count method (Chen *et al*., 2001).

### Preparation of roseophage DNA

Each phage was added into a 500 ml host culture (OD_600_ = 0.1∼0.2) with moi of 3, and incubated overnight. Phage lysates (10^10^−10^11^ phage particles ml^−1^) was mixed with 10 ml chloroform (2% v/v) and 20 g NaCl, and left on ice for 30 min before the cell debris was pelleted by centrifugation at 10 000 *g* for 30 min. The supernatant was mixed well with polyethylene glycol 8000 to a final concentration of 10% (w/v) and incubated overnight at 4°C. The phage particles were precipitated by centrifugation at 15 000 *g* for 30 min and then re-suspended in 10 ml of TM buffer (Tris-HCl 20 mM, MgSO_4_ 10 mM, pH 7.4). Polyethylene Glycol-concentrated phage lysates were overlaid onto a 10–50% iodixanol (OptiPrep, Sigma-Aldrich, MO, USA) gradient, and centrifuged for 2 h at 200 000 *g*, using a T-8100 rotor in a Sorvall Discovery 100S centrifuge. The visible viral band was extracted using a 18-gauge needle syringe and then dialysed twice in TM buffer overnight at 4°C. Purified phages were stored at 4°C in the dark. Phage DNA was extracted using the method described previously (Sambrook and Russell, 2001).

### Genome sequencing and analysis

To prepare DNA template for genome sequencing, purified phage DNA was amplified using Genomiphi V2 kit (GE Healthcare, Piscataway, NJ, USA) according to the manufacturer's protocol. The initial sequence segments were obtained by random PCR amplification of phage DNA using degenerate primer RP-1 (5′-ATHGAYGGNGAYATHCAY-3′) and RP-2 (5′-YTCRTCRTGNACCCANGC-3′). The PCR was performed in 50 μl volume containing 1× reaction buffer (Genescript, Scotch Plains, NJ, USA) with 1.5 mM MgCl_2,_ 100 μM of dNTPs, 50 pmol of each primer, 1 U Taq DNA polymerase (Genescript) and 10 ng phage DNA as templates. PCR program consists of an initial denaturing at 94°C for 2 min, followed by 30 cycles of denaturing at 94°C for 30 s, annealing at 48°C for 1 min and extension at 72°C for 1 min and a final extension at 72°C for 10 min. Multiple PCR amplicons could be obtained for both phages. The most dominant bands for each phage were excised and the DNAs were purified using gel purification kit (Qiagen, Valencia, CA, USA) and sequenced bi-directionally using the same primer set. The three fragments with unambiguous sequences are used as starting templates for primer walking. All the subsequent primer walking was done by using an automated sequencer ABI 310 (PE Applied Biosystems) in the Biological and Analytical Laboratory at the Center of Marine Biotechnology, UMBI. From each primer walking, unambiguous sequences were assembled together using AssemblyLIGN program (GCG, Madison). Open reading frames were predicted by using ORF Finder (http://www.ncbi.nlm.nih.gov/gorf/gorf.html) and GeneMarkS (Besemer and Borodovsky, 1999). Translated ORFs were compared with known protein sequences using blastp (Altschul *et al*., 1990). tRNA sequences were searched by using tRNAscan-SE (Lowe and Eddy, 1997). The countcodon program was used to determine codon usage (http://www.kazusa.or.jp/codon/countcodon.html).

Sequences alignment and phylogenetic analysis were performed using MacVector 7.2 program (GCG, Madison, WI). Jukes–Cantor distance matrix analysis was used to calculate the distances from the aligned sequences, and the neighbour-joining method was used to construct the phylogenetic tree.

### GOS database search

The amino acid sequence of N4-like DNA *pol* gene was searched against the GOS metagenomic database using blastp (Seshadri *et al*., 2007) (*E*-value < 10^−20^). The blast homologues of the DNA *pol* gene were then searched against the NCBI database, only the sequences closely related to N4 DNA *pol* were retained and other sequences closer to the bacterial DNA *pol* were not included.

### Nucleotide sequence accession number

The GenBank accession numbers assigned to the complete DSS3Φ2 and EE36Φ1 genomes are FJ591093 and FJ591094 respectively.

## Supporting information

*Fig. S1.* Genome comparison of DSS3Φ2 and EE36Φ1. Red arrow: DSS3Φ2 unique ORF, blue arrow: EE36Φ1 unique ORF, white arrow: shared ORF.

*Fig. S2.* Amino acid sequence alignment of four Motifs (T/DxxGR, A, B and C) between N4-like phages v-RNAPs and other T-7 supergroup podoviruses RNAPs. Residues that highlighted in red are identical in all these phages, Residues that highlighted in yellow are > 50% identical. The arrows indicate the conserved catalytic residues.

*Fig. S3.* Amino acid sequence comparison of Roseophage *ssb* gene and N4 *ssb* gene. The arrows indicate the catalytic residues in N4.

*Fig. S4.* Neighbour-joining tree constructed based on the aligned *rnr* family amino acid sequences. Sequences from DSS3Φ2 and EE36Φ1 are shown in bold. The scale bar represents 0.1 fixed mutations per amino acid position. Bootstrap = 1000.
