# Supplementary material for: Genome sequences of two novel phages infecting marine roseobacters
Source: Environ Microbiol. 2009 Aug;11(8):2055–64. doi: 10.1111/j.1462-2920.2009.01927.x (PMC2784036; doi:10.1111/j.1462-2920.2009.01927.x)
Supplement: Supplementary file 1 [file emi0011-2055-SD1.doc]

**SUPPLEMENTAL FIGURES**

**Genome sequences of two novel phages infecting marine roseobacters**

Yanlin Zhao ^1,2^, Kui Wang ^1^, Nianzhi Jiao ^2, *^, and Feng Chen ^1, *^,

1. Center of Marine Biotechnology, University of Maryland Biotechnology Institute, Baltimore, MD 21202, USA
2. State Key Laboratory of Marine Environmental Science, Xiamen University, Xiamen 361005, China

Fig. S1. Genome comparison of DSS3Φ2 and EE36Φ1. Red arrow: DSS3Φ2 unique ORF, blue arrow: EE36 Φ1 unique ORF, white arrow: shared ORF

Fig. S2. Amino acid sequence alignment of four Motifs (T/DxxGR, A, B and C) between N4-like phages v-RNAPs and other T-7 supergroup podoviruses RNAPs. Residues that highlighted in red are the identical in all these phages, Residues that highlighted in yellow are >50% identical. The arrows indicate the conserved catalytic residues.

Fig. S3. Amino acid sequence comparison of Roseophage *ssb* gene and N4 *ssb* gene. The arrows indicate the catalytic residues in N4.

Fig. S4. Neighbor-joining tree constructed based on the aligned *rnr* family amino acid sequences. Sequences from DSS3Φ2 and EE36Φ1 are shown in bold. The scale bar represents 0.1 fixed mutations per amino acid position. Bootstrap = 1,000.
